# The Influence of Heart Rate Variability Biofeedback on Cardiac Regulation and Functional Brain Connectivity

**DOI:** 10.3389/fnins.2021.691988

**Published:** 2021-06-29

**Authors:** Andy Schumann, Feliberto de la Cruz, Stefanie Köhler, Lisa Brotte, Karl-Jürgen Bär

**Affiliations:** ^1^Lab for Autonomic Neuroscience, Imaging and Cognition (LANIC), Department of Psychosomatic Medicine and Psychotherapy, Jena University Hospital, Jena, Germany; ^2^Department of Psychiatry and Psychotherapy, Jena University Hospital, Jena, Germany; ^3^Institute of Medical Psychology and Behavioral Immunobiology, Essen University Hospital, Essen, Germany

**Keywords:** autonomic nervous system, resting state functional connectivity, prefrontal cortex, insula, cingulate cortex

## Abstract

**Background:**

Heart rate variability (HRV) biofeedback has a beneficial impact on perceived stress and emotion regulation. However, its impact on brain function is still unclear. In this study, we aimed to investigate the effect of an 8-week HRV-biofeedback intervention on functional brain connectivity in healthy subjects.

**Methods:**

HRV biofeedback was carried out in five sessions per week, including four at home and one in our lab. A control group played *jump‘n’run games* instead of the training. Functional magnetic resonance imaging was conducted before and after the intervention in both groups. To compute resting state functional connectivity (RSFC), we defined regions of interest in the ventral medial prefrontal cortex (VMPFC) and a total of 260 independent anatomical regions for network-based analysis. Changes of RSFC of the VMPFC to other brain regions were compared between groups. Temporal changes of HRV during the resting state recording were correlated to dynamic functional connectivity of the VMPFC.

**Results:**

First, we corroborated the role of the VMPFC in cardiac autonomic regulation. We found that temporal changes of HRV were correlated to dynamic changes of prefrontal connectivity, especially to the middle cingulate cortex, the left insula, supplementary motor area, dorsal and ventral lateral prefrontal regions. The biofeedback group showed a drop in heart rate by 5.2 beats/min and an increased SDNN as a measure of HRV by 8.6 ms (18%) after the intervention. Functional connectivity of the VMPFC increased mainly to the insula, the amygdala, the middle cingulate cortex, and lateral prefrontal regions after biofeedback intervention when compared to changes in the control group. Network-based statistic showed that biofeedback had an influence on a broad functional network of brain regions.

**Conclusion:**

Our results show that increased heart rate variability induced by HRV-biofeedback is accompanied by changes in functional brain connectivity during resting state.

## Introduction

The heart is the central organ of the circulatory system that pumps blood through the arterial vessel network in order to provide oxygen for all vital organs. Although the activity of the heart is driven by an intrinsic pacemaker called sinoatrial node, it is additionally influenced by environmental demands. Body signals shape the workload of the heart in order to meet changing needs of the entire organism. The two peripheral branches of the autonomic nervous system (ANS), the parasympathetic and the sympathetic system, modulate the intrinsic activity of the cardiac pacemaker cells in the sinoatrial node. While the sympathetic branch is needed for an adequate stress response, parasympathetic or vagal activation reduces expenditure and promotes health. Thus, the heart rate mirrors the resulting homeostasis of an organism influenced by internal and external demands. It is, therefore, conceivable that a complex system is needed to orchestrate autonomic cardiac function.

On the basis of early animal experiments and lesion studies, Benarroch described the “central autonomic network” (CAN) including several regions of the forebrain, limbic system, and the brainstem (Benarroch, 1993). In a previous meta-analysis, we found that core nodes of the CAN have been consistently reported by modern neuroimaging studies on regulation of the ANS, i.e., the cingulate cortex, anterior insula, ventromedial prefrontal cortex (VMPFC), mediodorsal thalamus, amygdala, hypothalamus, etc (Beissner et al., 2013). Further reviews corroborate a cortico-limbic network, including VMPFC, cingulate cortex, insula, and amygdala, to drive central autonomic control (Thayer et al., 2012; Shoemaker et al., 2015).

As a more theoretical framework, Thayer and colleagues introduced the neurovisceral integration model that links cognitive and emotional states to autonomic function (Thayer and Lane, 2000). In their approach, the prefrontal cortex is the highest level of a hierarchical model with direct functional connections to limbic regions, i.e., the insula and cingulate (Thayer and Lane, 2000; Thayer et al., 2012). The limbic system is further connected via the amygdala to subcortical downstream regions such as hypothalamus and brainstem nuclei that determine parasympathetic and sympathetic modulation of the heart, at the lowest level of the model. According to this construct, the prefrontal cortex and its top-down control over subcortical structures has a pivotal role in heart rate regulation and relates sympathovagal balance to cognitive and emotional processing (Thayer et al., 2009).

Using magnetic resonance imaging and resting-state functional connectivity (RSFC) studies have corroborated the important role of the interaction between the medial prefrontal cortex and limbic regions in heart rate regulation (Sakaki et al., 2016; Kumral et al., 2019). In a recent publication, we compared RSFC patterns between groups of healthy individuals that differed in heart rate regulation. Our results indicated that subjects with slow heart rates have significantly increased RSFC in a functional network of several regions of the central autonomic and the sensorimotor system when compared to subjects with fast heart rate (de la Cruz et al., 2019). Interestingly, we observed an increased RSFC between the prefrontal cortex (VMPFC) and the anterior insula to be associated with slow heart rates.

Whereas heart rate is modulated by both branches of the autonomic nervous system, heart rate variability (HRV) is a marker of parasympathetic cardiac regulation. It is generally accepted that high variability of the heart rate is in many aspects health-promoting. Thus, lower levels of HRV have been associated with increased cardiovascular morbidity and mortality (Thayer and Lane, 2007). Hillebrand et al. (2013) reported that healthy subjects with diminished resting HRV have a 32–45% increased risk to suffer from a first cardiovascular event (Hillebrand et al., 2013). Furthermore, HRV is thought to be associated with cognitive performance and emotional well-being [see reviews (Forte et al., 2019; Mulcahy et al., 2019)]. High heart rate variability implies a flexible autonomic nervous system that adapts rapidly to changing demand which improves behavioral control (Porges, 1995, 2007). The brainstem seems to be the anatomical mediator between autonomic flexibility and central processes such as attention, emotion, and communication (Porges, 1995). Whether these correlational associations describe HRV as a consequence of central regulatory processes or as a prerequisite for effective regulation is still unclear (Mather and Thayer, 2018). In a recent opinion paper, Mather and Thayer (2018) proposed that oscillations in emotion-regulating networks can be enhanced by HRV biofeedback (Mather and Thayer, 2018). HRV biofeedback is a bio-behavioral intervention to augment vagal tone. It is based on the phenomenon of respiratory sinus arrhythmia that describes heart rate to increase during inhalation and to decrease during exhalation. Thus, subjects can modulate their heart rate and thereby their HRV by modifying their breathing pattern.

Several studies demonstrated a beneficial effect of biofeedback in psychiatric disorders such as depression (Gevirtz, 2013). A meta-analytic review of 24 studies, including 484 participants showed that HRV biofeedback reduces perceived stress and anxiety levels significantly (Goessl et al., 2017). Thus, it has been speculated that HRV biofeedback influences brain function. Its similarity to electrical vagal nerve stimulation has been focused in order to explain underlying physiological mechanisms (Lehrer and Gevirtz, 2014). As a method of treatment in patients with epilepsy or depression, a pulse generator that is implanted in the chest wall stimulates primarily afferent vagal fibers. Impulses reach the brainstem and influence areas in the forebrain that are involved in the regulation of emotions, cognitive and autonomic function such including the frontal cortex, amygdala, and insula (Henry, 2002; Nemeroff et al., 2006; Lehrer and Gevirtz, 2014).

In this study, we aimed to investigate the effect of HRV biofeedback on functional brain organization. We hypothesized that HRV biofeedback slows resting heart rate and increases its variability. As we assume that HRV is closely tied to fronto-limbic connectivity, we hypothesized increased functional connectivity between the VMPFC and core regions of the limbic system, especially the anterior insula and the cingulate cortex, after biofeedback intervention.

## Materials and Methods

### Study Group Formation

We investigated 32 healthy participants that completed this intervention study excluding seven subjects that dropped out due to illness (two control, one biofeedback group) or not adhering to the study protocol by skipping sessions (three biofeedback group). Subjects with similar age and same gender were paired and then randomly assigned to the intervention group and the control group. Fifteen participants performed a biofeedback training (seven males; eight females; age: 30 ± 8 years, 22–52 years, BMI: 24.4 ± 3.0 kg/m^2^). Seventeen participants completed a control intervention (eight males; nine females; age: 29 ± 10 years, 18–53 years, BMI: 24.4 ± 3.4 kg/m^2^). All participants were white Caucasian that we recruited from the local community via flyers and online advertisement. Pregnancy, the intensive pursuit of endurance sports, cardiovascular diseases (e.g., hypertension and diabetes), neurological disorders (e.g., migraine, epilepsy, and multiple sclerosis), or psychiatric disorders (e.g., depression, attention deficit hyperactivity disorder, and anxiety disorder) were held as exclusion criteria. All participants gave written informed consent to a protocol approved by the Ethics Committee of the medical faculty of the Friedrich-Schiller University Jena (# 5423-01/18) in accordance with the Declaration of Helsinki.

### Intervention Protocol

The intervention took 8 weeks in which participants of the biofeedback group performed an HRV biofeedback training. Five training sessions per week had to be conducted, including four sessions at home and one session at the laboratory, 5-min resting period and two training runs lasting 11 min with a short pause in between. Subjects in the control group played one of three different *jump’n’run* mobile games in sessions organized according to the same schedule as the biofeedback group.

During the intervention, heart rate was recorded using a sensor incorporated in a belt that was tied around the chest of the subject (H10/H7 Heart Rate Sensor; Polar Electro Oy, Kempele, Finland). Via Bluetooth, the application EliteHRV (Elite HRV LLC, 2017) collected data from the sensor, stored recordings and displayed heart rate. Participants in the control group recorded heart rate in the background while playing a mobile game. In the biofeedback group, heart rate oscillations were displayed on the screen of their smartphone as instantaneous visual feedback of heart rate. Participants were asked to adapt their breathing patterns in such a way to enhance heart rate oscillations, as described below. After each training session, we received raw data acquired during that session per email from participants of both groups. Thus, we were able to track the progress of the training throughout the intervention.

Magnetic resonance imaging (MRI) was conducted before the beginning of the training (T1) and after finishing the schedule (T2). One week prior to T1, an additional MRI session was planned to obtain participants’ habituation to the procedure (T0). Physiological signals and resting state scans were acquired simultaneously in order to assess cardiac autonomic function.

### HRV Biofeedback

In the biofeedback group, participants’ current heart rate (HR) was shown as an interpolated smoothed curve on their smartphone display. Their final goal was to maximize HR oscillations. For a detailed description of the intervention that was designed following the manual published by Lehrer et al. (2000) we refer to our previous publication that reports preliminary results from a subset of the current sample (Schumann et al., 2019).

In short, we estimated the individual resonance frequency which was then used as a pacer for participants’ breathing rhythm during the first 2 weeks of training. After that, the HR curve was displayed during the sessions for participants to breathe “in phase” with their HR curve by inhaling when HR ascended and exhaling when it descended. After another 3 weeks, participants were instructed to expand the amplitudes of the HR curve in order to maximize HRV for 3 weeks.

### MRI Data Acquisition

The data were collected on a 3T whole-body system equipped with a 12-element head matrix coil (MAGNETOM Prisma, Siemens Healthineers, Erlangen, Germany). Participants were instructed to keep their eyes open during the whole measurement and to move as little as possible. T2^∗^- weighted images were obtained using a multiband multislice GE-EPI sequence (TR = 484 ms, TE = 30 ms, FA = 90°, Multiband Factor = 8) with 56 contiguous transverse slices of 2.5 mm thickness covering the entire brain and including the lower brainstem. The matrix size was 78 × 78 pixels with an in-plane resolution of 2.5 × 2.5 mm^2^. A series of 1,900 whole-brain volume sets were acquired in one session (about 15 min). In one participant of the biofeedback group and in 2 control subjects we recorded 1,250 volumes only. High-resolution anatomical T1-weighted volume scans (MPRAGE) were obtained in a sagittal slice orientation (TR = 2,300 ms, TE = 3.03 ms, TI = 900 ms, FA = 9°, acquisition matrix = 256 × 256 × 192, acceleration factor PAT = 2) with an isotropic resolution of 1 mm^3^.

### Physiological Recordings and Analyses

We recorded finger pulse and respiratory movement during resting fMRI sessions using a MP150 (BIOPAC Systems Inc., Goleta, CA, United States). An optical finger pulse sensor was attached to the proximal phalanx of the index finger of the subject’s left hand. Pulse waves were extracted from the first derivative of the pulse signal due the rapid signal increase on pulse arrival. Automatic detections were visually inspected for missing peaks and artifacts. Inter-beat-interval (IBI) time series were calculated and analyzed by inhouse MATLAB scripts (R2016a, The MathWorks Inc., Natick, MA, United States). Finally, subject’s mean HR was computed, as well as global [standard deviation of heartbeat intervals (SDNN)] and short term [root mean square of successive heartbeat interval differences (RMSSD)] measures of HRV (Malik et al., 1996). Breathing rate (BR) was estimated as inverse of the average interval between respiratory maxima that were derived from the respiration signal. The quality of respiratory peak detection was also inspected for artifacts and corrected manually when necessary.

### Resting State Functional MRI Preprocessing

Data preprocessing was performed using AFNI^1^ and SPM12^2^. The first twenty images were discarded, allowing magnetization to reach a steady state. Physiological noise correction was performed by including four low-order Fourier time series to reduce artifacts synchronized with the respiratory cycle (Glover et al., 2000) and five respiration volumes per time (RVT) regressors that model slow blood oxygenation level fluctuations (Birn et al., 2008; Jo et al., 2010). The RVT regressors consisted of the RVT function and four delayed terms at 5, 10, 15, and 20 s (Birn et al., 2008).

Further preprocessing included realignment to the first volume using a rigid body transformation. For each participant, head movement was below 3 mm and 3°. Additional preprocessing steps were (i) removal of lineal and quadratic trends and of several sources of variance, i.e., head-motion parameter, CSF and white matter signal, (ii) temporal band-pass filtering, retaining frequencies in the 0.01–0.1 Hz band, and (iii) spatial smoothing using a Gaussian kernel of full-width half maximum of 6 mm. Extra-cerebral tissue was removed from the anatomical images using ROBEX (Iglesias et al., 2011), a learning-based brain extraction method trained on manually “skull-stripped” data from healthy subjects. These skull-stripped brains were aligned to the standard MNI 2-mm brain. Finally, functional images were registered to anatomical data and normalized to the MNI space by applying transformation parameters derived from the anatomical to MNI registration.

### Region of Interest and Functional Connectivity Analyses

Based on our hypothesis, a region of interest was defined in the VMPFC as the seed region for functional connectivity analyses. The VMPFC-ROI was drawn as a sphere of 10 mm radio centered at MNI-coordinates, *x* = 0, *y* = 44, *z* = −14, as defined in our previous study (de la Cruz et al., 2019).

To obtain functional connectivity maps, preprocessed resting-state fMRI signal was averaged over each voxel with the seed region and correlated against all voxels in the brain. The resulting Pearson correlation coefficients were converted to Fisher *z* statistics in order to produce a more normally distributed variable (de la Cruz et al., 2019).

The effect of biofeedback training was evaluated comparing VMPFC correlation maps at T1 to T2 (paired *t*-test). The effect of group (biofeedback vs. control) on RSFC changes between T1 and T2 was assessed using a two-sample *t*-test of z-map differences (T2-T1). Statistical results were thresholded with *p* < 0.005 uncorrected at voxel-level and family-wise error corrected (*p* < 0.05) at cluster level.

### Sliding-Window Analysis of Functional Connectivity and Heart Rate Variability

The correlation of functional connectivity changes with temporal changes of HRV was estimated at T1 using data from all participants (*N* = 30). In two subjects, quality of the pulse signal was not good enough to build a complete regressor. We calculated variability of heart rate (SDNN) and functional connectivity of the VMPFC ROI in sliding time windows of 45 s length (90 TR) with 50% overlap (45TR) (Chang et al., 2013). On the single-subject level, we performed a linear regression analysis of VMPFC RSFC maps and the HRV regressor. Contrast images were then passed into a one-sample *t*-test group analysis. The statistical map was thresholded at an uncorrected voxel-level significance of *p* < 0.005 and FWE corrected at cluster-level.

### Network Analysis of Resting State Functional MRI

In addition to the seed-based FC approach, we investigated significant between-group differences in the whole-brain network connectivity (connectome) using the network-based statistic approach (NBS) (Zalesky et al., 2010).

Individual connectivity matrices were generated extracting the mean time series from 260 independent anatomical ROIs, which were defined based on the coordinates from an extensively validated parcelation system provided by Power et al. (2011). Each ROI was modeled as 10 mm diameter sphere with a minimum distance of 10 mm between sphere centers, thus avoiding potential overlapping. In addition, we discarded short-distance correlations less than 20 mm since it might be affected by spatial smoothing or reslicing. A paired *t*-test design was then performed on each group separately by comparing T2 vs. T1. Here, components were identified using a primary component-forming threshold at *t* > 4.17. Permutation testing (10,000 permutations) was then applied to calculate FWE for every component previously identified. Results were considered significant for *p* < 0.05. NBS analysis was conducted using the Brain Connectivity Toolbox (Rubinov and Sporns, 2010).

## Results

### Temporal Co-variation of Prefrontal Connectivity and Heart Rate Variability

**FIGURE 1:**
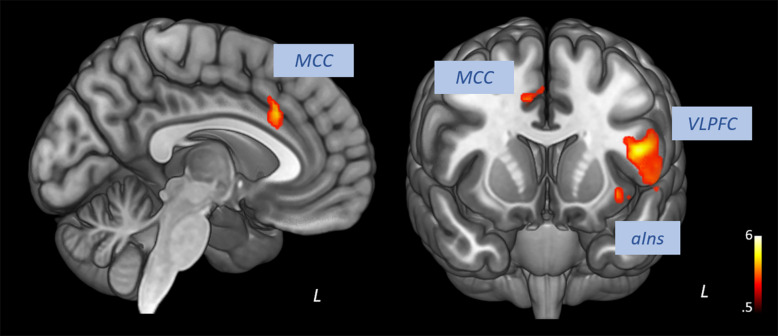
Correlation of heart rate variability changes with changes of functional connectivity of the prefrontal cortex (voxel-level: *p* < 0.005 uncorr., cluster-level: *p* < 0.05 FDR-corrected). MCC, middle cingulate cortex; VLPFC, ventral lateral prefrontal cortex; aIns, anterior insula.

Pooling data from participants of both groups prior to the intervention (T1), we aimed to corroborate the association of heart rate variability and connectivity of the prefrontal cortex irrespective of biofeedback. In sliding windows, we estimated synchronous changes of functional connectivity and SDNN that was extracted from physiological recordings during the scan. Individual *z*-maps of the correlation between those time series were tested for a significant temporal co-variation (one-sample *t*-test). Dynamic changes of HRV were correlated to changes of prefrontal connectivity, especially to the middle cingulate cortex, the insula, dorsal, and ventral lateral prefrontal regions (see Figure 1 and Table 1).

**TABLE 1:** Correlation of prefrontal connectivity changes with heart rate variability changes (voxel-level: *p* < 0.005 uncorr., cluster-level: *p* < 0.05 FDR-corrected).

Region	Left/Right	Cluster size	Brodmann’s area	MNI coordinates			*t*
				*x*	*y*	*z*	
Middle cingulate gyrus	R	205	32	14	6	40	5.46
	L		32	−4	22	32	4.49
Ventral lateral frontal gyrus	L	440	44	−48	12	14	5.21
Cerebellum	R	237		44	−44	−34	4.86
Dorsal lateral frontal gyrus	R	1,453	9/8	36	42	26	4.75
Insula	L	281	13	−36	20	−2	4.67
Ventral lateral frontal gyrus	L		47	−46	30	−4	4.54
Dorsal lateral frontal gyrus	L	303	10/9	−32	40	30	4.09
Occipital gyrus	R	201		16	−98	−4	3.72

### Effect of Biofeedback on Heart Rate Variability

As depicted in Table 2, resting heart rate during functional scans decreased after the biofeedback intervention by 3.9 beats/min (4.9%) and global HRV (SDNN) increased by 8.6 ms (18%). Short term (RMSSD) HRV as well as the breathing rate did not change significantly. The control intervention had no significant effect on any of these parameters.

**TABLE 2:** Changes of heart rate variability and breathing rate from before (T1) to after the intervention (T2) in the biofeedback and control group.

*Parameter*	*Biofeedback group*				*Control group*			
	T1	T2	T2-T1	Significance	T1	T2	T2-T1	Significance
*HR [min^–1^]*	70.1 ± 9.6	65.0 ± 8.4	−5.2 ± 7.3	*p* < 0.05	67.9 ± 8.1	69.6 ± 8.3	1.7 ± 7.9	n.s.
*SDNN [ms]*	54.4 ± 14.9	63.0 ± 18.5	8.6 ± 14.3	*p* < 0.05	68.9 ± 30.7	63.1 ± 27.7	−5.9 ± 27.5	n.s.
*RMSSD [ms]*	46.4 ± 18.3	53.9 ± 19.6	7.5 ± 19.4	n.s.	57.4 ± 29.3	51.8 ± 24.9	−5.6 ± 26.3	n.s.
*BR [bpm]*	16.0 ± 4.4	15.2 ± 2.8	−0.8 ± 3.6	n.s.	15.1 ± 3.1	15.2 ± 3.6	0.9 ± 4.4	n.s.

*Parameters were assessed during resting fMRI. T1, before intervention; T2, after intervention; HR, heart rate; RMSSD, root mean square of successive heart beat interval differences; SDNN, standard deviation of heart beat intervals; BR, breathing rate; n.s., not significant.*

### Effect of Biofeedback on Functional Connectivity of the Prefrontal Cortex

**FIGURE 2:**
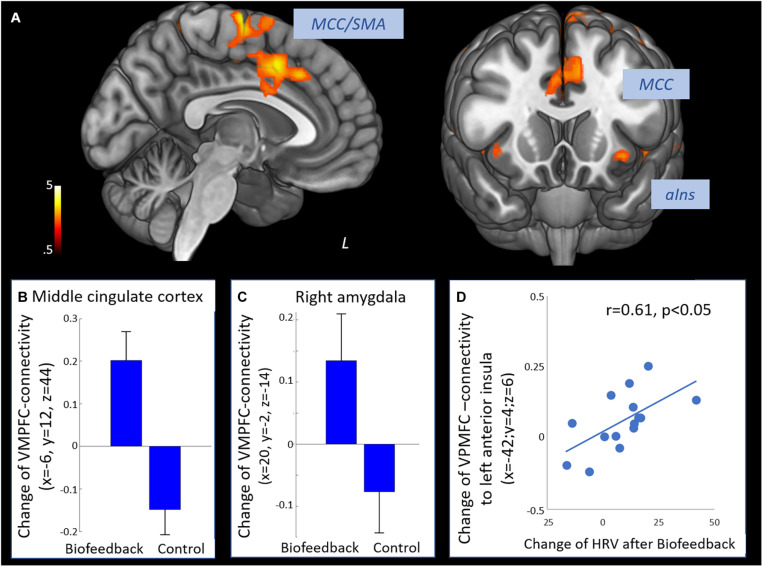
Effect of biofeedback on functional connectivity of the prefrontal cortex. **(A)** Positive interaction contrast time × group revealing higher increases of functional connectivity of the VMPFC from T1 to T2 in the biofeedback group compared to the control group (voxel-level: *p* < 0.005 uncorr., cluster-level: *p* < 0.05 FDR-corrected). **(B)** Change of connectivity from T1 to T2 in the middle cingulate cortex. **(C)** Change of VMPFC-connectivity from T1 to T2 in the right amygdala. **(D)** Change of VMPFC-connectivity from T1 to T2 in the left anterior insula was correlated to the increase of HRV (Pearson *r* = 0.61, *p* < 0.05). SMA, supplementary motor area; MCC, middle cingulate cortex; aIns, anterior insula.

The time × group interaction contrast revealed increased connectivity between the VMPFC and the middle cingulate cortex, the supplementary motor area, dorsal and ventral lateral prefrontal regions, posterior and anterior insula and the right amygdala in the biofeedback when compared to the control group (see Figure 2 and Table 3). The change of prefrontal connectivity to the left anterior insula (*x* = −42, *y* = 4, *z* = 6) was correlated to the change of HRV in the biofeedback group (*r* = 0.61, *p* < 0.05, Figure 2D).

**TABLE 3:** Interaction effect time **×** group on changes of functional connectivity of the prefrontal cortex (voxel-level: *p* < 0.005 uncorr., cluster-level: *p* < 0.05 FDR-corrected).

Region	Left/	Cluster size	Brodmann’s area	MNI coordinates			*t*
	Right			*x*	*y*	*z*	
Superior parietal Lobe	L	7,369	5	−20	−42	62	5.07
Middle cingulate gyrus	R		32	4	16	38	3.48
Putamen	R	1,760		32	−2	−2	4.53
Amygdala	R			20	−2	−14	3.76
Insula	R			48	−8	−4	3.73
Dorsal lateral frontal gyrus	L	1,206	10	−40	42	24	4.35
Ventral lateral frontal gyrus			10/46	−44	54	−4	4.11
Superior temporal gyrus	L	2,248	22/41	−60	14	−4	4.28
Insula	L		13	−42	4	6	3.39
Dorsal lateral frontal gyrus	L	359	6	−52	2	40	4.15

### Effect of Biofeedback on Functional Network Organization

**FIGURE 3:**
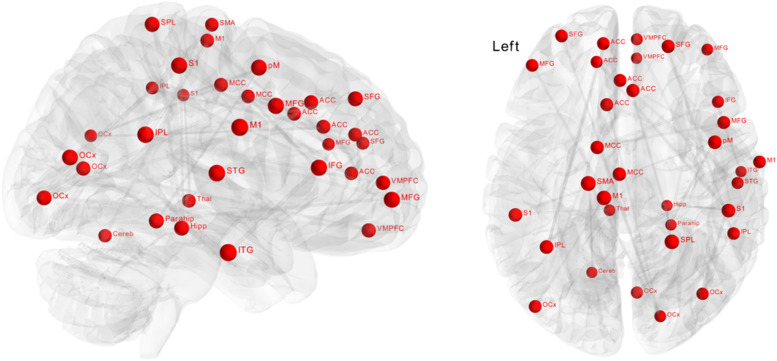
Effect of biofeedback on functional connectivity matrices using network-based statistics (NBS). The graph shows nodes with significantly (*p* = 0.048) higher connectivity after biofeedback intervention. These connections formed a single connected network with 34 nodes and 33 edges.

Significantly greater positive functional connectivity was observed after the biofeedback intervention in a network of 34 nodes and 33 edges (Figure 3, *p* = 0.048) revealed by the NBS analysis. Nodes within this network were located in central autonomic regions, i.e., amygdala, ventromedial prefrontal cortex, anterior cingulate cortex, but also in visual, temporal and sensorimotor regions with a large number of intra-hemispheric functional connections. There were no significant changes in the control group.

## Discussion

Our results demonstrate decreased heart rates after HRV biofeedback training. Additionally, we found enhanced functional connectivity of the prefrontal cortex to a number of important cortical regions. A wide functional network of brain regions seems to be affected by biofeedback intervention.

Biofeedback has been demonstrated to restore autonomic dysfunction in patients with cardiovascular disorders (Gevirtz, 2013). For example, in a group of patients with coronary artery disease, an HRV biofeedback intervention increased resting HRV, reduced blood pressure, and also decreased hostility behavior (Lin et al., 2015). As high HRV has been linked to better response inhibition and emotion regulation (Ruiz-Padial et al., 2003; Williams et al., 2015, 2017), HRV biofeedback might enhance regulatory brain regions (Mather and Thayer, 2018). Core brain regions that are involved in the experience and perception of emotions are the anterior insula, the amygdala, and the cingulate cortex (Lindquist et al., 2012). The prefrontal cortex has regulatory control over these regions and determines the cognitive processing and interpretation of feelings (Ochsner and Gross, 2005; Dillon and Pizzagalli, 2007).

Considering the CAN described by Benarroch, 1993), a wide range of brain regions crucial for emotional processing are also involved in autonomic control. As emotional arousal is closely tied to autonomic responses, it is not surprising that their neural representations overlap (Critchley, 2005, 2009). Cognitive regulation via the prefrontal cortex can neutralize emotional affective experiences and decrease accompanying physiological arousal (Jackson et al., 2000; Gross, 2002). Our current results show that even at rest, regulatory control of the medial prefrontal cortex over limbic regions is closely related to HRV changes. We found that dynamic functional connectivity between the ventromedial prefrontal cortex and the anterior insula, the cingulate cortex and the amygdala correlated with the time course of HRV.

The HRV biofeedback intervention increased prefrontal functional connectivity, especially to the anterior insula, middle cingulate cortex, amygdala, thalamus, and lateral prefrontal regions. According to the neurovisceral integration model, prefrontal control over the cingulate cortex and the insula reflects the highest level of a top-down regulatory chain involving the amygdala, hypothalamus, and the brainstem to modulate cardiac activity (Nikolin et al., 2017). Sympatho-excitatory subcortical circuits are under tonic inhibitory control by the PFC (Amat et al., 2005). For example, the amygdala, which has outputs to autonomic, endocrine, and other physiological regulatory systems and is activated during threat and uncertainty, is under tonic inhibitory control via GABAergic projections from the PFC (Davidson, 2000; Thayer, 2006). Thus, in normal modern life, the sympatho-excitatory preparation for flight and fight is tonically inhibited. However, under conditions of uncertainty or threat, the PFC becomes hypoactive which is associated with disinhibition of sympatho-excitatory circuits that are essential for physical and mental responses. Similarly, it has been postulated that psychopathological states such as anxiety or depression are associated with prefrontal dysfunction leading to poor habituation to novel neutral stimuli or unbalanced threat information processing (Rogers et al., 2004; Holmes et al., 2005). As a consequence, sympatho-excitatory circuits become disinhibited in these conditions leading to abnormal emotional processing as well as to an autonomic imbalance (Makovac et al., 2016; Ottaviani et al., 2016).

The enhancement of interactions between specific brain regions might underly the beneficial influence of HRV biofeedback on emotion regulation. Network-based statistics revealed that the connectivity in a wide network of regions was influenced by biofeedback with nodes located in the central autonomic network, but also in the visual and sensorimotor system. In a former network analysis, we compared groups of healthy subjects with different resting heart rates (de la Cruz et al., 2019), that showed subjects with slow heart rate have higher connectivity in a network comprising mainly sensorimotor and occipital regions. Comparing this network with the results of the current analysis, it is conspicuous that more regions of the frontal lobe are included in the network affected by biofeedback such as the cingulate and prefrontal regions. There are several studies showing that those regions are involved in heart rate regulation and may play an important role in the top-down processes related to voluntary modulation of heart rate (Thayer et al., 2012). For instance, non-invasive stimulation of the dorsolateral PFC has been demonstrated to reduce HR and enhance HRV (Makovac et al., 2017).

How biofeedback of a peripheral autonomic signal influences functional brain organization is still unclear (Lehrer and Gevirtz, 2014; Mather and Thayer, 2018). By adapting breathing in order to maximize heart rate oscillations, participants “exercise” principle vagal reflexes, especially the baroreflex (Lehrer et al., 2003). The baroreflex is one of the most powerful mechanisms of short-term heart rate modulation. Pressure sensors called baroreceptors detect changes of blood pressure and initiate adaptation of cardiovascular function. Immediate influences on heart rate are vagally mediated via autonomic centers in the brainstem (Schumann et al., 2017).

By augmenting vagal afferent input, HRV biofeedback is thought to stimulate those cardiovagal brainstem nuclei similar to direct electrical stimulation (Lehrer and Gevirtz, 2014). Vagal nerve stimulation acts on the central autonomic network, and the limbic system by modulating vagal afferent activity (Henry, 2002). The nucleus of the solitary tract (NTS) is the primary integration center of sensory information from the periphery, including discharge patterns of baroreceptors and lung stretch receptors, with projections to noradrenergic and serotonergic neuromodulatory systems (Chamberlin, 2004; Saper, 2011). Using fMRI, it has been demonstrated that vagal nerve stimulation increases activity of the NTS and enhances its functional connectivity to the cingulate cortex and anterior insula (Garcia et al., 2017; Sclocco et al., 2019).

## Conclusion

Our data suggest that HRV biofeedback increases HRV and decreases heart rate. Changes of autonomic cardiac regulation are accompanied by enhanced functional connectivity of the prefrontal cortex to core regions of emotional and cognitive processing.

## Data Availability Statement

The datasets presented in this study can be found in online repositories. The names of the repository/repositories and accession number(s) can be found below: https://openneuro.org/datasets/ds003357.

## Ethics Statement

The studies involving human participants were reviewed and approved by Ethics Committee of the Medical Faculty of the Friedrich-Schiller University Jena (# 5423-01/18). The patients/participants provided their written informed consent to participate in this study.

## Author Contributions

AS contributed to analysis and interpretation of the data and preparing the manuscript. FC contributed to analysis and interpretation of the data. SK contributed to acquisition and analysis of the data. LB contributed to acquisition and preprocessing of the data. K-JB contributed to study conception, preparing the manuscript, and critical revision. All authors contributed to the article and approved the submitted version.

## Conflict of Interest

The authors declare that the research was conducted in the absence of any commercial or financial relationships that could be construed as a potential conflict of interest.

## Footnotes

1 https://afni.nimh.nih.gov/

2 http://www.fil.ion.ucl.ac.uk/spm
